# Thrombotic Thrombocytopenic Purpura Masked by Severe Preeclampsia: A Case Report

**DOI:** 10.7759/cureus.74851

**Published:** 2024-11-30

**Authors:** Dalal Mahmoud Alabdulmohsen, Jehad Nizar Alhashim

**Affiliations:** 1 Internal Medicine, College of Medicine, King Faisal University, Al Ahsa, SAU; 2 Hematology and Oncology, King Saud Medical City, Riyadh, SAU

**Keywords:** aquired ttp, case report, hematologic emergencies, preeclampsia, pregnancy complications, thrombotic thrombocytopenic purpura

## Abstract

Thrombotic thrombocytopenic purpura (TTP) is an exceptionally rare complication during pregnancy and even rarer when it coincides with severe preeclampsia in the same index pregnancy. We report the case of a 36-year-old female who presented with confusion at 38 weeks of gestation. Although her signs and symptoms strongly suggested severe preeclampsia, she was expected to make a full recovery after an emergency C-section. However, her failure to recover and persistent deterioration indicated the need for further investigations into the cause. Once her blood smear showed fragmented cells, plasma exchange treatment was initiated immediately without waiting for the results of the ADAMST13 activity test, which later showed almost undetectable levels. Ultimately, the patient achieved a full recovery, and her platelet count as well as ADAMST13 levels continued to improve upon 12 weeks of follow-up with no further treatment needed. Proper clinical judgment and a cooperative multidisciplinary team were key elements in managing this case.

## Introduction

Thrombotic thrombocytopenic purpura (TTP) constitutes a life-threatening hematologic emergency. Despite the availability of treatment modalities, a significant mortality risk of approximately 20% persists [1]. In the absence of prompt diagnosis and appropriate intervention, mortality rates associated with TTP can escalate to a critical level exceeding 90% [1,2]. The clinical presentation of TTP encompasses a triad of findings: profound thrombocytopenia, microangiopathic hemolytic anemia, and a range of ischemic dysfunctions primarily affecting the brain, heart, and kidneys. This syndrome arises from a marked deficiency (<10% of the normal level) of ADAMTS13, a plasma metalloprotease essential for cleaving von Willebrand factor (VWF) forms within the vasculature. The critical impairment in the breakdown of VWF leads to the formation of ultra-large multimers, ultimately causing end-organ ischemia and the characteristic clinical picture [3].

TTP is a rare condition, with epidemiological data suggesting an approximate incidence rate of only two new cases per million population yearly [4]. Most diagnosed patients are female adults, with 12% of them being pregnant women [4,5]. The occurrence of TTP in pregnancy is a special situation that poses many clinical challenges and thus should be managed by a collaborative multidisciplinary team. One challenge is the need for early and accurate distinction between preeclampsia and HELLP (hemolysis, elevated liver enzymes, and low platelets) syndrome, as they may present in a similar pattern initially [3,6]. These conditions, however, are more prevalent in pregnancy compared to TTP, which may result in the latter being easily overlooked by obstetric providers [7].

While preeclampsia improves and resolves after delivery of the infant and placenta, pregnant women with TTP require different management and further lifesaving interventions [7]. This case describes a pregnant woman who presented to the emergency room (ER) with confusion. However, solely managing her diagnosed preeclampsia failed to achieve a complete resolution.

## Case presentation

A 36-year-old pregnant woman at 38 weeks of gestation, with no documented history of chronic medical illness, presented to the emergency room (ER) with confusion. This was her first pregnancy, and she had no previous episodes of similar complaints. She had been compliant with her antenatal appointments, and her pregnancy was uncomplicated before this event.

On physical examination, her body temperature was normal at 36.7°C, oxygen saturation was 98%, and pulse rate was 93 beats per minute. However, her blood pressure was significantly elevated at 170/118 mmHg. She scored 14 out of 15 on the Glasgow Coma Scale, indicating mild confusion. Her fundal height was consistent with her gestational age, and further examination of her chest, heart, and abdomen revealed no significant findings apart from lower limb edema.

Table 1 displays the patient’s laboratory results at the time of admission. Her lab workup revealed low hemoglobin (7.57 g/dL), a low platelet count (21 × 10³/μL), and elevated white blood cells (17 × 10³/μL). Her reticulocyte count was high (12%), and lactate dehydrogenase (LDH) levels were markedly elevated at 1278 IU/L. Although liver enzymes were normal, total bilirubin and indirect bilirubin levels were high at 27 mg/dL and 22 mg/dL, respectively.

**Table 1:** Laboratory test results on admission

Lab tests	Results	Reference ranges
Hemoglobin (Hgb)	7.57 (g/dL)	12–16 (g/dL)
Red blood cell (RBC)	2.47 (10^6^/μL)	4–5.4 (10^6^/μL)
Hematocrit	23.20%	36%–54%
Mean corpuscular volume (MCV)	94 (fL)	76–96 (fL)
White blood cell (WBC)	17 (10^3^/μL)	4–11 (10^3^/μL)
Neutrophils	89.20%	40%–80%
Lymphocytes	7.40%	20%–40%
Platelets (plt)	21 (10^3^/μL)	150–450 (10^3^/μL)
Lactate dehydrogenase (LDH)	1278 (IU/L)	126–266 (IU/L)
Reticulocyte count	12%	0.5%–1.5%
Alanine aminotransferase (ALT)	39 (U/L)	10–40 (U/L)
Aspartate aminotransferase (AST)	28 (U/L)	10–35 (U/L)
Total bilirubin	27 (mg/dL)	0.3–1.2 (mg/dL)
Direct bilirubin	5 (mg/dL)	0–0.4 (mg/dL)

Urine analysis of the patient showed proteinuria. At this point, the patient was diagnosed with severe preeclampsia. The patient was not in active labor. A vaginal examination showed no cervical dilation, and cardiotocography (CTG) showed no contractions in addition to normal fetal heart rate and variability (class 1). She was rushed to the operating room for an emergency cesarean section, where she received platelet transfusions and was started on IV magnesium sulfate, ultimately delivering a healthy baby with no need for NICU admission and no fetal or maternal obstetric complications. After her delivery, her main complaint did not resolve, as she was still confused for another three days. So, a hematology consultation was requested subsequently.

**Figure 1:**
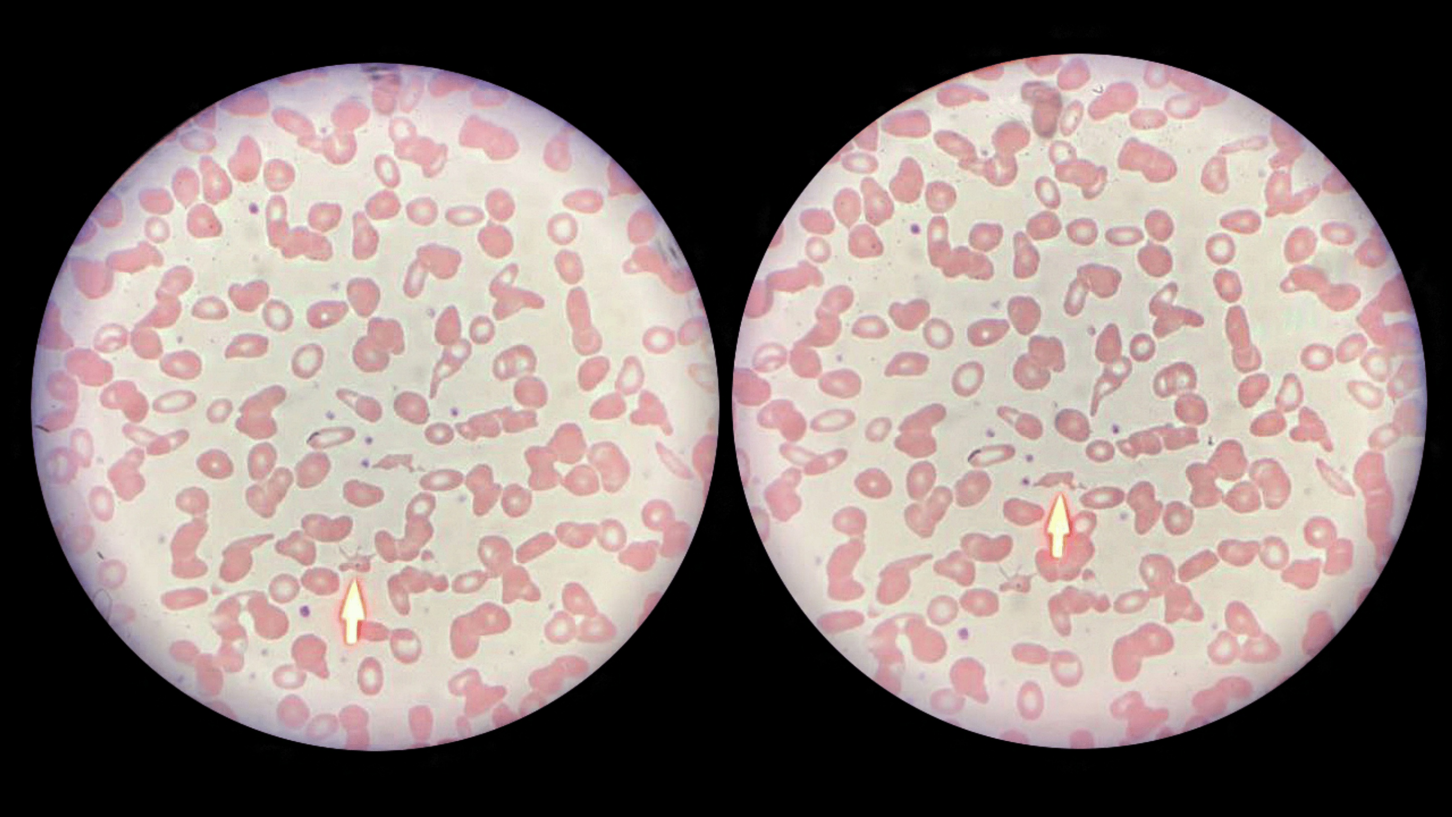
The patient’s blood smear showing fragmented cells Arrows point to the fragmented cells.

A peripheral blood smear was ordered, which showed 4% schistocytes (Figure 1) and large platelets. Her PLASMIC score was 7 (corresponding to a high probability of TTP), and a blood sample was sent for an urgent ADAMST13 level. In the meantime, the patient was started on 1000 mg of daily pulse steroids (methylprednisolone) for three days and therapeutic plasma exchange with 1.5 plasma volumes. After two sessions of plasma exchange on two consecutive days, her confusion resolved, and her platelet levels started to increase.

**Figure 2:**
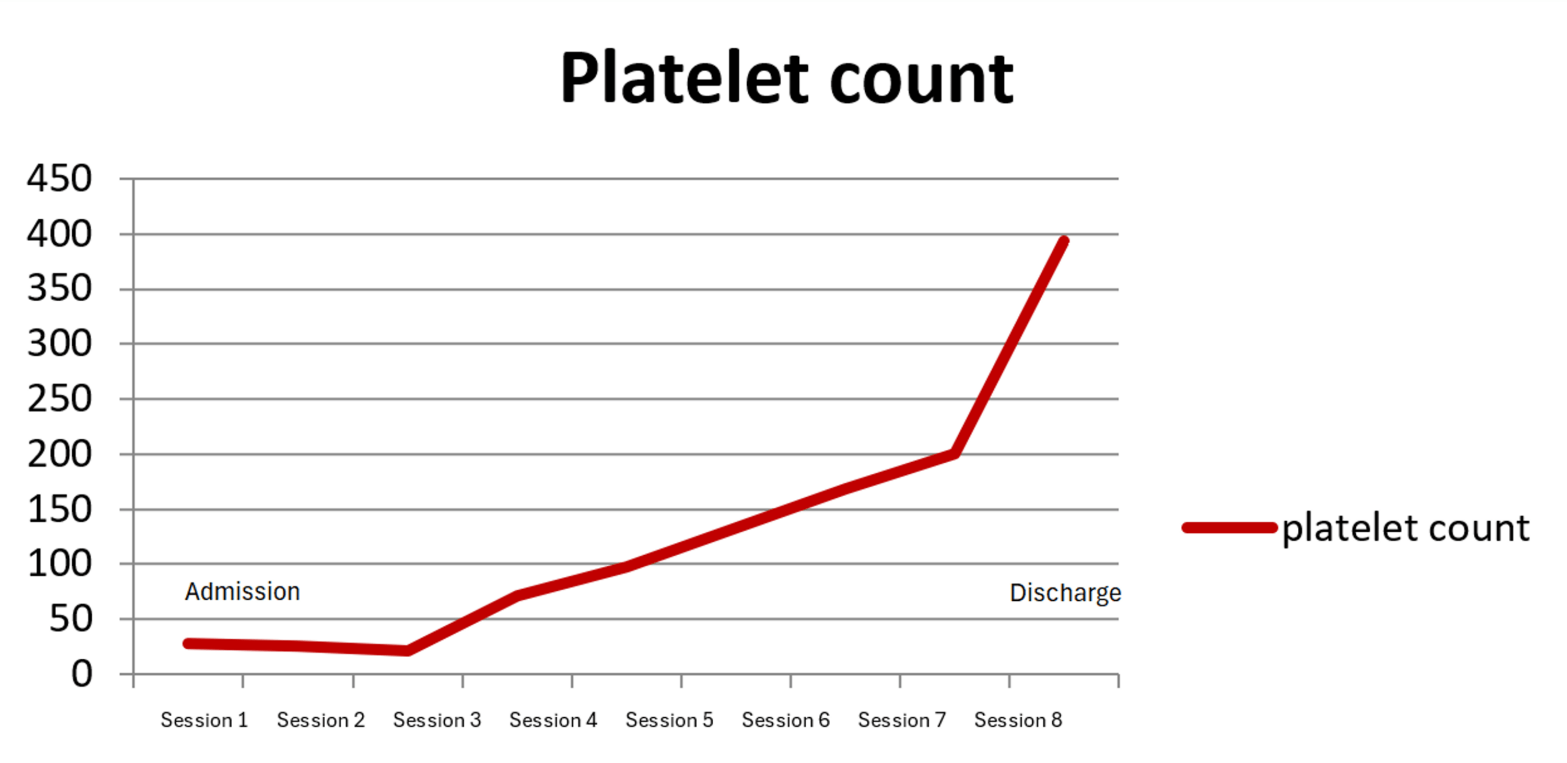
The patient’s platelet count over eight sessions of treatment Count × 10^3^ is on the Y-axis, and the plasma exchange session number is on the X-axis.

The patient received a total of eight sessions of plasma exchange, which led to her platelet count normalizing to exactly 200 x 10^3^/μL. These sessions were conducted on eight consecutive days and discontinued abruptly after day 8. Results revealed that her ADAMST13 activity was severely low (0.1%; normal range: 60%-130%) at the time of her presentation. The patient was discharged after scheduling a follow-up appointment in another two weeks. At the follow-up, her new platelet count was 394 x 10^3^/μL, with ADAMST13 new levels recorded as 50%. She was concluded to have had peripartum TTP. Without any further treatment, and one more week later, her ADAMST13 levels continued to rise, and her last result was 76%. Figure 2 shows the platelet counts over one month of plasma exchange. Rituximab was not needed for the management of this case.

## Discussion

TTP is an extremely rare complication of pregnancy, with a prevalence of 0.004% among all pregnancies [8]. The condition can mimic severe preeclampsia, but the simultaneous occurrence of both conditions appears in less than 20% of TTP cases during pregnancy, significantly elevating the risk of fetal and maternal complications compared to TTP alone [9]. Although TTP and severe preeclampsia have different etiologies, their presence may overlap with a confusing presentation in a minority of cases, necessitating a high index of suspicion, accurate identification, and urgent management to avoid catastrophic outcomes [10].

During pregnancy, there is a physiological increase in VWF concentrations, which inversely affects ADAMTS13 activity levels and leads to a progressive decrease, particularly in late pregnancy. This decrease in ADAMTS13 activity levels is usually not clinically significant. However, in some cases where its levels become undetectable, pregnancy itself can be a triggering factor for an acute peripartum episode of TTP rather than the typical immunological etiology seen in acquired TTP patients. In these rare cases, the acquired deficiency is not immune-mediated and does not depend on immunosuppression for treatment [10].

In the reported case, a patient in her last weeks of pregnancy presented to the ER with hypertension, proteinuria, and confusion. As the symptoms of severe preeclampsia are similar to those of TTP, and preeclampsia is much more common, the patient's condition was initially treated as severe preeclampsia, and differential diagnoses (such as TTP) were overlooked. However, since the main pathology behind preeclampsia is related to placental and maternal vascular dysfunction, the failure of recovery after giving birth clearly suggested an alternative diagnosis [11]. The persistence of symptoms after delivery, the significant decrease in platelet count, the neurological manifestation, and the high PLASMIC score were the first definite clues to consider the alternate diagnosis of TTP. The presence of schistocytes in the peripheral blood smear strongly indicated the need for further investigations to confirm the diagnosis. While pre-eclampsia is a common cause of fragmented cells, TTP and other causes require different management and, therefore, are significant to identify. However, since waiting for the results of specific investigations may take a long time, and delays in treatment further increase the risk of complications, management with plasma exchange is best to be initiated based on clinical judgment [12,13]. In our case, the patient improved drastically with plasma exchange sessions and made a full recovery. While we believe her TTP was mostly induced by pregnancy itself, a gene study might be helpful to evaluate the possibility of congenital TTP and help with pre-pregnancy counseling for her future pregnancies [14].

## Conclusions

This case report highlights the need to never disregard differential diagnoses in the presence of a more likely diagnosis without properly excluding life-threatening possibilities. Furthermore, it encourages multidisciplinary approaches in the management of complex cases where two difficult conditions coincide. In our case, the difficulty in diagnosis is owed to the non-specific presentation, the overlap in clinical manifestations of the two conditions, and the rarity of their co-occurrence. A high index of suspicion and decent judgment are key for making lifesaving decisions.
